# Association between the postoperative glycemic variability and mortality after craniotomy: a retrospective cohort study and development of a mortality prediction model

**DOI:** 10.3389/fendo.2025.1613662

**Published:** 2025-07-17

**Authors:** Yuanshuo Ge, Guangdong Wang, Yun Huang, Yaxin Zhang

**Affiliations:** ^1^ Jinzhou Medical University,The Third Clinical Medical College, Jinzhou, Liaoning, China; ^2^ Department of Respiratory and Critical Care Medicine, First Affiliated Hospital of Xi’an Jiaotong University, Xi’an, Shanxi, China; ^3^ Department of International Medical Center, First People’s Hospital of Foshan, Foshan, Guangdong, China; ^4^ Department of Neurology, Xiamen Humanity Hospital, Fujian Medical University, Xiamen, Fujian, China

**Keywords:** glycemic variability, craniotomy, mortality, retrospective cohort study, machine learning prediction model

## Abstract

**Background:**

Glycemic variability (GV), typically quantified by the coefficient of variation (CV) and the root mean square of successive differences (rMSSD), has been recognized as a potential predictor of poor outcomes in critically ill patients. However, its prognostic value in neurosurgical populations remains unclear. This study investigated the association between postoperative GV and mortality following craniotomy.

**Methods:**

We retrospectively analyzed 1,969 adult ICU patients who underwent cranial surgery. GV was measured using both CV and rMSSD calculated from blood glucose values during the ICU stay. The primary outcome was 28-day all-cause mortality; the secondary outcome was 90-day mortality. Multivariable Cox regression, restricted cubic splines, threshold effect analysis, and mediation analysis via blood urea nitrogen (BUN) were conducted. A Random Survival Forest (RSF) model was developed using machine learning and interpreted with SHAP values.

**Results:**

Higher GV, as reflected by both elevated CV and rMSSD, was independently associated with increased 28-day and 90-day mortality (CV per 10-unit HR: 1.20; rMSSD per 10-unit HR: 1.02; all P < 0.01). BUN partially mediated the association between GV and mortality. GV outperformed traditional clinical scores (SOFA, GCS, CCI) in ROC analysis (CV AUC = 0.72). The RSF model achieved an AUC of 0.841 and identified GV metrics as top predictors.

**Conclusions:**

Postoperative glycemic variability, assessed by CV and rMSSD, is an independent and modifiable predictor of short- and mid-term mortality following craniotomy. These findings highlight the clinical importance of GV in postoperative risk stratification and support its integration into neurosurgical critical care.

## 1 Introduction

Neurosurgical procedures are among the most complex and resource-intensive interventions in modern medicine, frequently employed to treat acute neurological emergencies such as traumatic brain injury, intracerebral hemorrhage, and space-occupying lesions (1, 2). Globally, an estimated 22.6 million individuals require neurosurgical care annually due to neurological conditions or injuries, with approximately 13.8 million necessitating surgical intervention (3). Among these procedures, craniotomies are considered particularly hazardous and impose a substantial economic burden (4). A study of high-risk craniotomy patients reported a 6-month mortality rate of 21%, with 43% experiencing poor outcomes—including death and severe disability (2, 5). The early postoperative period following craniotomy is particularly hazardous, characterized by rapid physiological changes and a significantly increased risk of mortality due to secondary brain injury caused by metabolic and inflammatory disturbances (6, 7). The development of cost-effective and simple biomarkers and mortality risk assessment models is urgently needed to improve postoperative management and outcomes.

Significant glycemic fluctuations are frequently observed in patients following neurosurgical procedures (8), primarily driven by neuroendocrine stress responses, perioperative inflammation, and iatrogenic factors such as glucose-containing infusions (9, 10). Increasing evidence suggests that such glycemic variability (GV) may exacerbate secondary brain injury through mechanisms including oxidative stress, endothelial dysfunction, mitochondrial impairment, and activation of neuroinflammatory cascades (11, 12). Both *in vitro* and human studies have demonstrated that GV induces more pronounced oxidative and endothelial injury than sustained hyperglycemia (13, 14). Notably, a large multicenter study found that GV was a stronger predictor of ICU mortality than mean glucose levels (15). Recent investigations have also linked GV to increased mortality in patients with atrial fibrillation and coronary artery disease (16, 17). However, its prognostic value in patients undergoing neurosurgical procedures remains poorly defined.

To quantify postoperative GV, we employed two complementary metrics: the coefficient of variation (CV) and the root mean square of successive differences (rMSSD) between consecutive glucose measurements (16). In this study, we aimed to evaluate the association between postoperative GV—measured by CV and rMSSD—and short-term and mid-term mortality in patients undergoing craniotomy. By characterizing the prognostic implications of GV in this high-risk population, we sought to identify modifiable risk markers that could inform postoperative management, improve clinical outcomes, and support the development of a mortality prediction model for neurosurgical patients that may be used in routine clinical practice.

## 2 Materials and methods

### 2.1 Research design

The MIMIC-IV v3.1 database is a freely accessible critical care database that includes de-identified data from ICU patients. This version was released on October 11, 2024, it contains ICU admission records spanning from 2008 to 2022, which is the full period currently available with structured clinical data. The database includes detailed information on demographics, vital signs, laboratory results, medications, diagnoses, and clinical outcomes, enabling large-scale retrospective clinical research. For this study, author Ge (ID: 13547277) followed the required data use protocols and was responsible for extracting the data from the MIMIC-IV v3.1 database, ensuring compliance with privacy and ethical guidelines.

The patient inclusion flow for this study is outlined as follows. Initially, 2,743 patients who were admitted to the ICU for the first time following cranial surgery were identified. After applying the inclusion criteria, 774 patients were excluded. Exclusion criteria included age under 18 years (n=0), ICU stay duration of less than 24 hours (n=380), and fewer than 3 blood glucose measurements (n=394). The remaining 1,969 patients constituted the final analysis cohort. This cohort was then subdivided into two groups based on survival status at 28 days: 1,713 patients survived, and 256 patients did not survive (**Supplementary Figure S1**).

### 2.2 Data gathering

Data were extracted from the MIMIC-IV v3.1 database using PostgreSQL software, focusing on first-day ICU admission data from patients who underwent cranial surgery, identified by ICD-coded procedures including craniotomy, cerebral or meningeal tissue resection, ventricular drainage or shunting, dural repair or replacement, and other major intracranial operations performed via open or percutaneous approaches. The collected data included patient demographics, vital signs, clinical scores, comorbidities, laboratory test results, treatments, and clinical events. Specifically, demographic information such as age, gender, race, and weight were recorded, along with vital signs including heart rate, blood pressure, respiratory rate, temperature, and oxygen saturation (SpO2). Clinical scores such as Sequential Organ Failure Assessment (SOFA), Glasgow Coma Scale (GCS), and Charlson Comorbidity Index (CCI) were also included, alongside information on comorbidities including myocardial infarction, heart failure, chronic pulmonary disease, cancer, acute kidney injury (AKI), sepsis, hyperlipidemia, diabetes, and hypertension. Laboratory results recorded included red blood cell count (RBC), white blood cell count (WBC), platelet count, sodium, potassium, calcium, international normalized ratio (INR), prothrombin time (PT), activated partial thromboplastin time (PTT), blood urea nitrogen (BUN), creatinine, and urine output. Treatment data comprised the use of epinephrine, norepinephrine, neuroblockers, insulin, statins, ondansetron, Proton Pump Inhibitors (PPI), propofol, and mannitol. Clinical events, such as length of hospital stay, ICU stay, hospital mortality, ICU mortality, and 90-day hospital mortality, were also collected.

### 2.3 Calculated variables and clinical outcomes

GV was assessed using two complementary metrics calculated over the entire ICU stay: the CV and the rMSSD. CV reflects overall glucose dispersion and is calculated using the formula: CV = (σ/μ)×100. where σ is the standard deviation of all blood glucose measurements and μ is the mean blood glucose level, expressed as a percentage. To evaluate short-term glycemic fluctuations, we also calculated rMSSD, which captures dynamic changes between consecutive glucose values. The formula is: rMSSD = sqrt[(1/(N–1))×Σ(BG_i_–BG_i−1_)²]. where BG_i_–BG_i−1_ represent two consecutive blood glucose readings, and N is the total number of measurements. This approach provides a time-sensitive indicator of short-term glycemic variability.

The primary outcome of this study was 28-day all-cause mortality, and the secondary outcome was 90-day all-cause mortality during the hospital stay.

### 2.4 Statistical methods

Descriptive statistics were performed for all participants. Continuous variables were expressed as either mean ± standard deviation (SD) for data following a normal distribution, or as median and interquartile range (IQR) for data with skewed distributions. To assess group differences, categorical variables were analyzed using the chi-squared test. For continuous data, the Student’s t-test was applied for normally distributed variables, while the Mann–Whitney U test was utilized for variables with skewed distributions. Variables with more than 15% missing data were excluded from the analysis. For variables with less than 15% missingness, multiple imputation was performed to handle missing values (**Supplementary Table S1**).

### 2.5 Analysis of the association between GV and clinical outcomes

Kaplan-Meier (KM) curves were used to explore the relationship between CV, rMSSD, and clinical outcomes, based on quartiles of CV and rMSSD. The log-rank test was used for group comparisons. Multivariable Cox proportional hazards regression was conducted to investigate the association between CV, rMSSD, and clinical outcomes, adjusting for demographic factors, comorbidities, and treatment measures. Restricted cubic splines (RCS) with three knots were employed to explore potential non-linear relationships between CV, rMSSD, and clinical outcomes. Threshold effect analysis was performed to identify inflection points for CV and rMSSD, determining the values that may represent turning points in the association with clinical outcomes. Interaction tests were conducted to evaluate potential interactions between CV, rMSSD, and other clinical variables, including demographic characteristics, comorbidities, and treatments. Mediation analysis was performed to investigate whether BUN acted as a mediator in the relationship between CV, rMSSD, and clinical outcomes. Receiver operating characteristic (ROC) curves were constructed to assess the diagnostic performance of CV and rMSSD in predicting clinical outcomes, with area under the curve (AUC) used to quantify accuracy.

### 2.6 Prediction model methodology

The original dataset was split into a 70% training set and a 30% validation set. All variables in the training set were included in feature selection using both Lasso and Boruta methods for clinical outcomes. However, for the sake of model simplicity, only the SOFA and CCI were considered as clinical scores in the feature selection process, while other clinical scores were excluded. This decision was made to ensure the predictive model remained simple and interpretable. The important features identified by LASSO and Boruta were then intersected to form a final set of features for model training. The models, including Cox Proportional Hazards Model (CoxPH), Classification and Regression Trees (CART), Gradient Boosting Machine (GBM), Ridge Regression (Ridge), Elastic Net (ENet), Neural Network (NN), Random Survival Forest (RSF), and Extreme Gradient Boosting (XGBoost), were trained using these selected features.

During the training process, grid search was employed to optimize model parameters. After training, the model’s performance was evaluated on the validation set using performance metrics such as the ROC curve, decision curve analysis, and calibration curve. The optimal model was further interpreted using SHAP values to explain the contributions of individual features. Finally, the best-performing model was deployed on a web platform for practical use.

The statistical analyses were conducted using R software (version 4.4.2), and statistical significance was set at a two-sided P-value of less than 0.05.

## 3 Results

### 3.1 Baseline characteristics

**Table 1** shows significant differences between the 28-day survival and non-survival groups for several variables with P-values < 0.05. CV and rMSSD were particularly noteworthy, with both showing significant differences between the two groups. Additionally, respiratory rate, temperature, SOFA score, GCS, CCI, and urine output also demonstrated significant variations. Comorbidities such as congestive heart failure, malignant cancer, AKI, sepsis, diabetes, and hypertension had p-values < 0.05. Laboratory results for INR, PT, PTT, BUN, creatinine, and platelet count also showed significant differences. Treatment use, including epinephrine, norepinephrine, insulin, ondansetron, propofol, and mannitol, was significantly higher in the non-survival group with P-values < 0.05.

**Table 1:** Baseline characteristics of postoperative cranial surgery patients.

Characteristic	Overall N = 1,969	28d-survival N = 1,713	28d-non-survival N = 256	P-value
Demographics				
Age (year)	63.60 (50.18, 74.16)	62.57 (49.31, 73.11)	70.61 (58.46, 79.61)	**<0.001**
Gender, n (%)				0.947
Female	896 (46%)	780 (46%)	116 (45%)	
Male	1,073 (54%)	933 (54%)	140 (55%)	
Race, n (%)				<0.001
Other^&^	692 (35%)	559 (33%)	133 (52%)	
White	1,277 (65%)	1,154 (67%)	123 (48%)	
Weight (Kg)	77.50 (65.90, 90.90)	77.30 (66.00, 90.90)	78.55 (64.90, 90.70)	0.943
Vital signs				
Heart rate (bmp)	79.00 (68.00, 92.00)	79.00 (68.00, 91.00)	79.50 (68.00, 94.00)	0.209
SBP (mmHg)	133.00 (119.00, 147.00)	132.00 (119.00, 147.00)	135.50 (118.00, 153.00)	0.103
DBP (mmHg)	69.00 (60.00, 78.00)	69.00 (60.00, 78.00)	69.00 (60.00, 79.00)	0.699
Respiratory rate (bmp)	17.00 (14.00, 20.00)	17.00 (14.00, 20.00)	19.00 (16.00, 22.00)	**<0.001**
Temperature (°C)	36.72 (36.44, 37.06)	36.72 (36.44, 37.06)	36.61 (36.33, 37.06)	**0.040**
SpO2 (%)	99.00 (97.00, 100.00)	99.00 (97.00, 100.00)	100.00 (98.00, 100.00)	**<0.001**
Clinical scores				
SOFA	1.00 (0.00, 1.00)	0.00 (0.00, 1.00)	1.00 (0.00, 3.00)	**<0.001**
GCS	15.00 (14.00, 15.00)	15.00 (14.00, 15.00)	15.00 (15.00, 15.00)	**0.032**
CCI	4.00 (2.00, 6.00)	4.00 (2.00, 6.00)	5.00 (3.00, 7.00)	**<0.001**
Comorbidities (%)				
Myocardial infarction, n (%)	103 (5%)	89 (5%)	14 (5%)	0.855
Congestive heart failure, n (%)	139 (7%)	104 (6%)	35 (14%)	**<0.001**
Chronic pulmonary disease, n (%)	240 (12%)	203 (12%)	37 (14%)	0.235
Malignant cancer, n (%)	357 (18%)	331 (19%)	26 (10%)	**<0.001**
AKI, n (%)	1,303 (66%)	1,085 (63%)	218 (85%)	**<0.001**
Sepsis, n (%)	891 (45%)	708 (41%)	183 (71%)	**<0.001**
Hyperlipidemia, n (%)	568 (29%)	482 (28%)	86 (34%)	0.072
Diabetes, n (%)	366 (19%)	293 (17%)	73 (29%)	**<0.001**
Hypertension, n (%)	1,125 (57%)	939 (55%)	186 (73%)	**<0.001**
Laboratory test				
RBC (10^9^/L)	3.97 (3.53, 4.37)	3.99 (3.56, 4.38)	3.77 (3.22, 4.25)	**<0.001**
WBC (10^9^/L)	12.10 (9.00, 15.40)	12.10 (9.00, 15.30)	12.10 (9.20, 15.75)	0.330
Platelet (10^9^/L)	217.00 (172.00, 268.00)	218.00 (176.00, 269.00)	194.00 (137.50, 266.00)	**<0.001**
Glucose CV	14.97 (9.69, 21.70)	14.15 (8.90, 20.53)	20.99 (15.45, 30.05)	**<0.001**
Glucose rMSSD	23.00 (14.80, 37.00)	21.65 (14.00, 34.56)	33.28 (22.20, 54.75)	**<0.001**
Sodium (mmol/L)	140.00 (137.00, 142.00)	140.00 (137.00, 142.00)	139.00 (136.00, 142.00)	0.073
Potassium (mmol/L)	3.90 (3.60, 4.30)	3.90 (3.60, 4.20)	3.90 (3.60, 4.40)	0.530
Calcium (mg/dL)	8.50 (8.10, 8.90)	8.50 (8.10, 8.90)	8.60 (8.10, 9.00)	0.682
INR	1.10 (1.10, 1.20)	1.10 (1.10, 1.20)	1.20 (1.10, 1.30)	**<0.001**
PT (S)	12.50 (11.60, 13.60)	12.40 (11.60, 13.40)	13.05 (12.10, 14.70)	**<0.001**
PTT (S)	26.80 (24.40, 29.40)	26.50 (24.30, 29.10)	28.00 (25.10, 31.40)	**<0.001**
BUN (mg/dL)	14.00 (10.00, 19.00)	14.00 (10.00, 19.00)	17.00 (13.00, 23.50)	**<0.001**
Creatinine (mg/dL)	0.80 (0.70, 1.00)	0.80 (0.70, 1.00)	0.90 (0.70, 1.20)	**<0.001**
Urine output (mL)	2,013.00 (1,375.00, 2,865.00)	2,050.00 (1,425.00, 2,875.00)	1,757.50 (1,140.00, 2,760.00)	**0.002**
Treatments				
Epinephrine, n (%)	10 (1%)	6 (0%)	4 (2%)	**0.031**
Norepinephrine, n (%)	159 (8%)	105 (6%)	54 (21%)	**<0.001**
Neuroblock, n (%)	33 (2%)	23 (1%)	10 (4%)	**0.007**
Insulin, n (%)	990 (50%)	820 (48%)	170 (66%)	**<0.001**
Statin, n (%)	427 (22%)	360 (21%)	67 (26%)	0.062
Ondansetron, n (%)	1,009 (51%)	910 (53%)	99 (39%)	**<0.001**
PPI, n (%)	193 (10%)	162 (9%)	31 (12%)	0.183
Propofol, n (%)	862 (44%)	663 (39%)	199 (78%)	**<0.001**
Mannitol, n (%)	239 (12%)	164 (10%)	75 (29%)	**<0.001**
Events				
Los hospital (day)	8.47 (4.68, 17.52)	8.61 (4.72, 18.03)	7.77 (4.06, 14.95)	**0.001**
Hospital Mortality, n (%)	217 (11%)	14 (1%)	203 (79%)	**<0.001**
Los ICU (day)	3.74 (2.10, 8.52)	3.52 (2.03, 8.01)	5.75 (2.86, 10.66)	**<0.001**
ICU Mortality, n (%)	150 (8%)	4 (0%)	146 (57%)	**<0.001**
90-day hospital Mortality, n (%)	343 (17%)	89 (5%)	254 (99%)	**<0.001**

^&^Includes patients recorded as Asian, Black or African American, Hispanic or Latino, Native American, or Other/Unknown in the original database.

SOFA, Sequential organ failure assessment; GCS, Glasgow Coma Scale; CCI, Charlson Comorbidity Index; SpO2, Oxygen saturation; SBP, Systolic blood pressure; DBP, Diastolic blood pressure; AKI, Acute kidney injury; WBC, White blood cell count; RBC, Red blood cell count; Platelet, Platelet count; CV, coefficient of variation; rMSSD, root mean square of successive differences; INR, International normalized ratio; PPI, Proton Pump Inhibitor; MV, Mechanical Ventilation; CRRT, Continuous renal replacement therapy.

Values in bold are statistically significant (p < 0.05).

### 3.2 Association between GV and 28-day and 90-day all-cause mortality

**Table 2** presents the multivariable Cox regression analysis for CV and rMSSD with 28-day and 90-day all-cause mortality in Model 3, which is fully adjusted for age, gender, race, weight, CCI, diabetes, respiratory rate, WBC, platelet, INR, insulin, and propofol use. The variables selected for this multivariable Cox regression model were based on univariate Cox regression (**Supplementary Table S2**), the Boruta algorithm, and clinical expert recommendations.

**Table 2:** Multivariable cox regression analysis of blood glucose fluctuations and mortality risk.

Variables	Model 1		Model 2		Model 3	
	HR (95%CI)	*P*	HR (95%CI)	*P*	HR (95%CI)	*P*
28-day hospital Mortality						
Glucose CV per 10 units	1.26 (1.20, 1.32)	**<0.001**	1.24 (1.18, 1.30)	**<0.001**	1.20 (1.14, 1.27)	**<0.001**
Glucose CV quantile						
Q 1	1.00 (Reference)		1.00 (Reference)		1.00 (Reference)	
Q 2	3.56 (1.92, 6.60)	**<0.001**	3.47 (1.87, 6.44)	**<0.001**	3.03 (1.63, 5.62)	**<0.001**
Q 3	6.30 (3.50, 11.33)	**<0.001**	5.77 (3.20, 10.40)	**<0.001**	4.90 (2.71, 8.86)	**<0.001**
Q 4	10.46 (5.90, 18.53)	**<0.001**	9.17 (5.16, 16.27)	**<0.001**	7.72 (4.32, 13.81)	**<0.001**
P for trend		**<00.001**		**<0.001**		**<0.001**
Glucose rMSSD per 10 units	1.03 (1.01, 1.04)	**<0.001**	1.02 (1.01, 1.03)	**<0.001**	1.02 (1.01, 1.03)	**0.004**
Glucose rMSSD quantile						
Q 1	1.00 (Reference)		1.00 (Reference)		1.00 (Reference)	
Q 2	2.27 (1.36, 3.81)	**0.002**	2.18 (1.30, 3.66)	**0.003**	1.92 (1.14, 3.23)	**0.014**
Q 3	3.76 (2.32, 6.10)	**<0.001**	3.40 (2.09, 5.51)	**<0.001**	2.88 (1.77, 4.69)	**<0.001**
Q 4	5.98 (3.76, 9.54)	**<0.001**	5.26 (3.29, 8.39)	**<0.001**	4.64 (2.86, 7.54)	**<0.001**
P for trend		**<0.001**		**<0.001**		**<0.001**
90-day hospital Mortality						
Glucose CV per 10 units	1.24 (1.19, 1.30)	**<0.001**	1.22 (1.16, 1.27)	**<0.001**	1.19 (1.13, 1.25)	**<0.001**
Glucose CV quantile						
Q 1	1.00 (Reference)		1.00 (Reference)		1.00 (Reference)	
Q 2	2.01 (1.31, 3.11)	**0.002**	2.01 (1.30, 3.10)	**0.002**	1.80 (1.16, 2.78)	**0.008**
Q 3	3.55 (2.38, 5.31)	**<0.001**	3.32 (2.22, 4.97)	**<0.001**	2.95 (1.96, 4.43)	**<0.001**
Q 4	5.71 (3.88, 8.40)	**<0.001**	5.06 (3.43, 7.47)	**<0.001**	4.41 (2.97, 6.56)	**<0.001**
P for trend		**<0.001**		**<0.001**		**<0.001**
Glucose rMSSD per 10 units	1.03 (1.02, 1.04)	**<0.001**	1.02 (1.01, 1.03)	**<0.001**	1.02 (1.01, 1.03)	**0.002**
Glucose rMSSD quantile						
Q 1	1.00 (Reference)		1.00 (Reference)		1.00 (Reference)	
Q 2	1.79 (1.20, 2.68)	**0.004**	1.76 (1.18, 2.63)	**0.006**	1.57 (1.05, 2.35)	**0.027**
Q 3	2.71 (1.86, 3.94)	**<0.001**	2.48 (1.71, 3.62)	**<0.001**	2.21 (1.52, 3.23)	**<0.001**
Q 4	4.33 (3.03, 6.19)	**<0.001**	3.79 (2.65, 5.43)	**<0.001**	3.27 (2.24, 4.76)	**<0.001**
P for trend		**<0.001**		**<0.001**		**<0.001**

Model 1 (Crude): Unadjusted.

Model 2: Adjusted for age, gender, race, and weight.

Model 3: Further adjusted based on Model 2, with additional covariates including CCI, diabetes, respiratory rate, WBC, platelet count, INR, insulin use, and propofol administration.

HR, hazard ratio; CI, confidence interval; CCI, Charlson Comorbidity Index; WBC, white blood cell count; CV, coefficient of variation; rMSSD, root mean square of successive differences; INR, international normalized ratio.

Values in bold are statistically significant (p < 0.05).

For 28-day hospital mortality, CV per 10 units was significantly associated with increased mortality risk (HR: 1.20, 95% CI: 1.14–1.27, P < 0.001). The highest quantile of CV (Q4) showed a strong association with mortality when compared to the lowest quantile (Q1) (HR: 7.72, 95% CI: 4.32–13.81, P < 0.001). For rMSSD, each 10-unit increase was significantly associated with increased mortality risk (HR: 1.02, 95% CI: 1.01–1.03, P = 0.004). The highest quantile of rMSSD (Q4) also showed a strong association with mortality when compared to Q1 (HR: 4.64, 95% CI: 2.86–7.54, P < 0.001).

For 90-day hospital mortality, CV per 10 units remained significantly associated with an increased risk (HR: 1.19, 95% CI: 1.13–1.25, P < 0.001). The highest quantile of CV (Q4) showed a strong association with mortality when compared to Q1 (HR: 4.41, 95% CI: 2.97–6.56, P < 0.001). Similarly, each 10-unit increase in rMSSD was significantly associated with higher mortality risk (HR: 1.02, 95% CI: 1.01–1.03, P = 0.002). The highest quantile of rMSSD (Q4) showed a strong association with mortality compared to Q1 (HR: 3.27, 95% CI: 2.24–4.76, P < 0.001).

**Figure 1:**
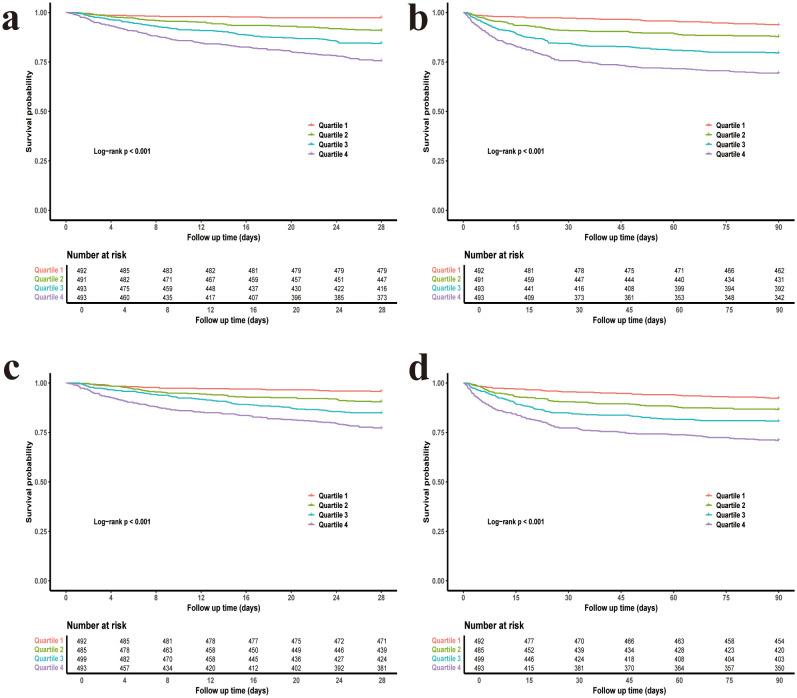
Kaplan–Meier survival curves for 28-day and 90-day mortality stratified by glycemic variability quartiles. **(a)** CV and 28-day mortality. **(b)** CV and 90-day mortality. **(c)** rMSSD and 28-day mortality. **(d)** rMSSD and 90-day mortality. CV, coefficient of variation; rMSSD, root mean square of successive differences.

In the Kaplan-Meier curve analysis, the highest quantile (Q4) for both CV and rMSSD showed the highest mortality rates compared to Q1, and the log-rank P-value for both variables was < 0.001, indicating that with the increase in blood glucose fluctuations, the risk of mortality significantly rises (**Figure 1**).

### 3.3 Nonlinear relationship between GV and 28-day and 90-day all-cause mortality

**Figure 2:**
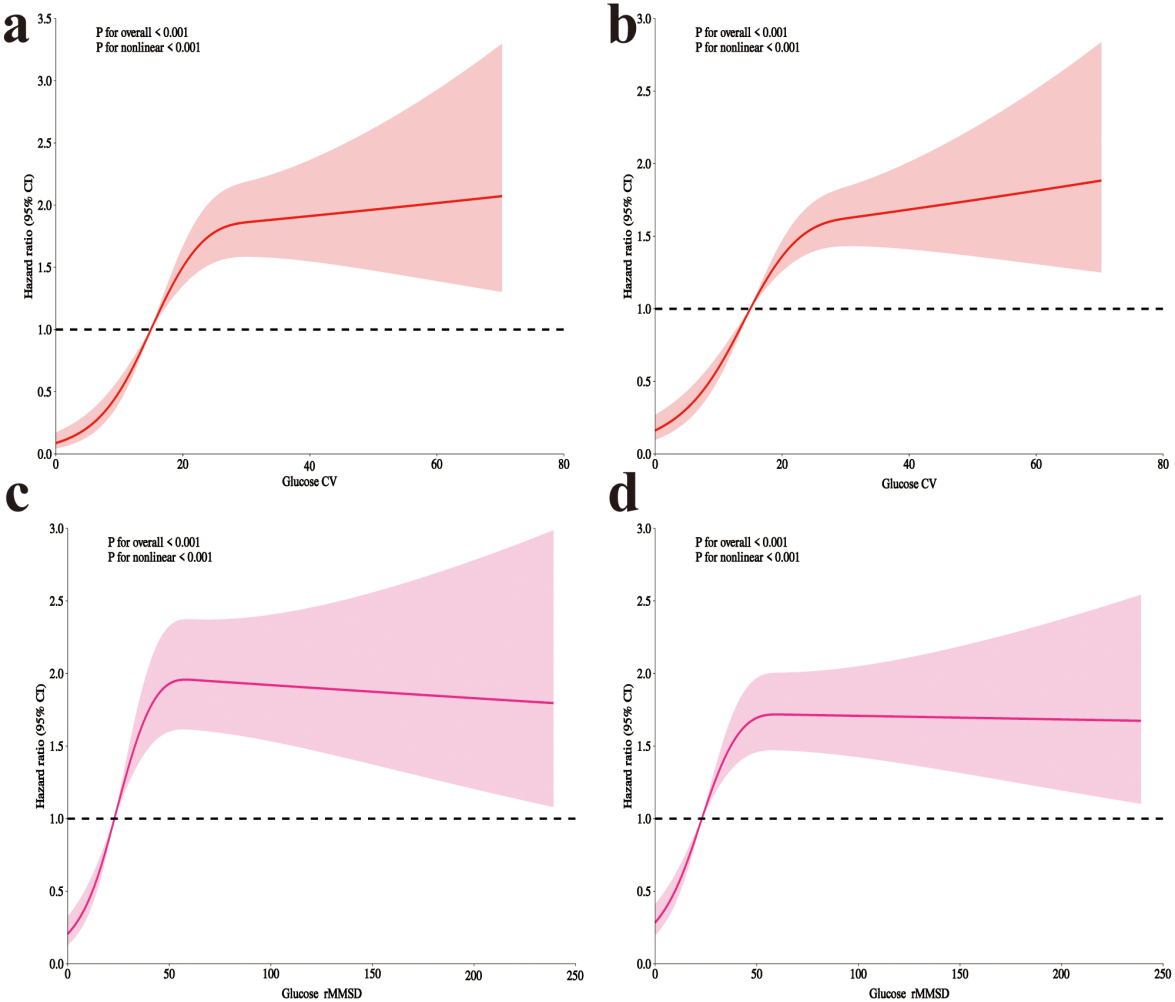
RCS analysis of glycemic variability and mortality in postoperative craniotomy patients. **(a)** 28-day mortality with glucose CV; **(b)** 90-day mortality with CV; **(c)** 28-day mortality with rMSSD; **(d)** 90-day mortality with rMSSD. RCS, Restricted cubic spline; CV, coefficient of variation; rMSSD, root mean square of successive differences.

After adjusting for all covariates, RCS analysis revealed a significant overall association between both CV and rMSSD with clinical outcomes (P for overall < 0.001 for both). The analysis also identified significant nonlinear relationships between both CV and rMSSD with clinical outcomes (P for nonlinear < 0.001 for both) (**Figure 2**).

**Table 3** presents the threshold effect analysis for CV and rMSSD. For 28-day mortality, each 10-unit increase in CV was associated with a HR of 1.20 (P < 0.001), with an inflection point at 20.5: hazard increased significantly below this threshold (HR 3.56, P < 0.001), but not above (HR 1.03, P = 0.547). For rMSSD, each 10-unit increase was associated with an HR of 1.02 (P = 0.004), with a significant increase below 43.8 (HR 1.57, P < 0.001), but not above (HR 0.99, P = 0.446). All likelihood ratio tests were significant (P < 0.001). For 90-day mortality, each 10-unit increase in CV was associated with an HR of 1.19 (P < 0.001), with an inflection point at 22.1: hazard increased significantly below (HR 2.50, P < 0.001), but not above (HR 1.02, P = 0.755). For rMSSD, the HR per 10-unit increase was 1.02 (P = 0.002), with a significant increase below 44.1 (HR 1.44, P < 0.001), and no difference above (HR 0.99, P = 0.619). All likelihood tests remained significant (P < 0.001).

**Table 3:** Threshold effect analysis of blood glucose fluctuations on mortality risk.

Outcome	HR (95% CI)	*P*
28-day hospital Mortality		
Glucose CV per 10 units		
Model 1 Fitting model by standard linear regression	1.20 (1.14, 1.27)	**<0.001**
Model 2 Fitting model by two-piecewise linear regression		
Inflection point	20.5	
20.5	3.56 (2.31, 5.47)	**<0.001**
≥20.5	1.03 (0.94, 1.13)	0.547
P for likelihood test		**<0.001**
Glucose rMSSD per 10 units		
Model 1 Fitting model by standard linear regression	1.02 (1.01, 1.03)	**0.004**
Model 2 Fitting model by two-piecewise linear regression		
Inflection point	43.8	
<43.8	1.57 (1.35, 1.82)	**<0.001**
≥43.8	0.99 (0.96,1.02)	0.446
P for likelihood test		**<0.001**
90-day hospital Mortality		
Glucose CV per 10 units		
Model 1 Fitting model by standard linear regression	1.19 (1.13, 1.25)	**<0.001**
Model 2 Fitting model by two-piecewise linear regression		
Inflection point	22.1	
<22.1	2.50 (1.88, 3.33)	**<0.001**
≥22.1	1.02 (0.92, 1.12)	0.755
P for likelihood test		**<0.001**
Glucose rMSSD per 10 units		
Model 1 Fitting model by standard linear regression	1.02 (1.01, 1.03)	**0.002**
Model 2 Fitting model by two-piecewise linear regression		
Inflection point	44.1	
<44.1	1.44 (1.27, 1.63)	**<0.001**
≥44.1	0.99 (0.97, 1.02)	0.619
P for likelihood test		**<0.001**

HR, Hazard Ratio; CI, Confidence Interval; CV, coefficient of variation; rMSSD, root mean square of successive differences.

Values in bold are statistically significant (p < 0.05).

### 3.4 Interaction analysis of GV and clinical variables on mortality risk

**Figure 3:**
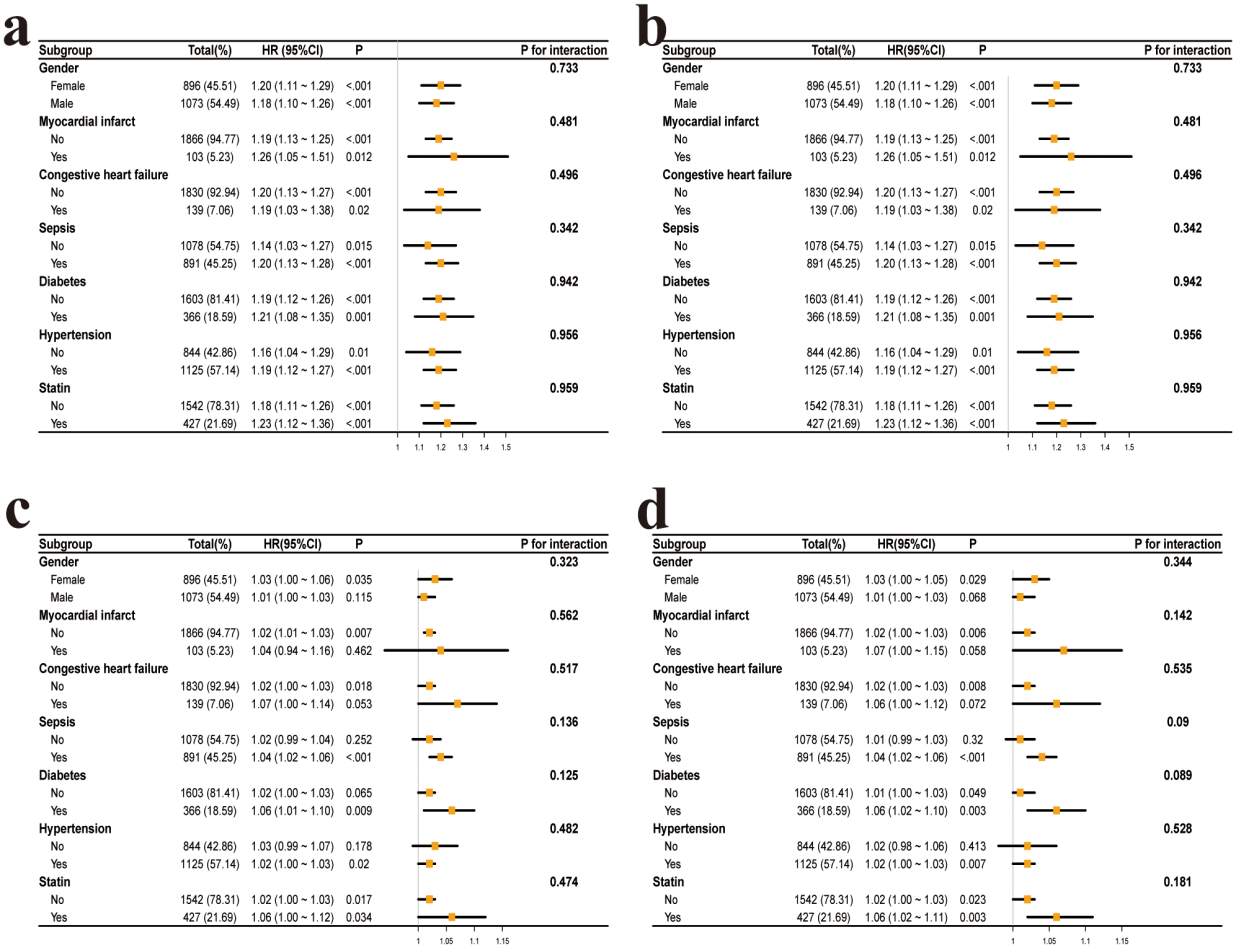
Subgroup analysis of the association between glycemic variability and mortality in postoperative craniotomy patients. **(a)** CV and 28-day all-cause mortality. **(b)** CV and 90-day all-cause mortality. **(c)** rMSSD and 28-day all-cause mortality. **(d)** rMSSD and 90-day all-cause mortality. CV, coefficient of variation; rMSSD, root mean square of successive differences.

After adjusting for all covariates, interaction analysis was performed based on the following subgroups: gender (female, male), myocardial infarction (yes, no), congestive heart failure (yes, no), sepsis (yes, no), diabetes (yes, no), hypertension (yes, no), and statin use (yes, no). The results showed that none of the interaction p-values were significant (**Figure 3**).

### 3.5 Mediation analysis of GV on mortality risk through BUN

**Figure 4:**
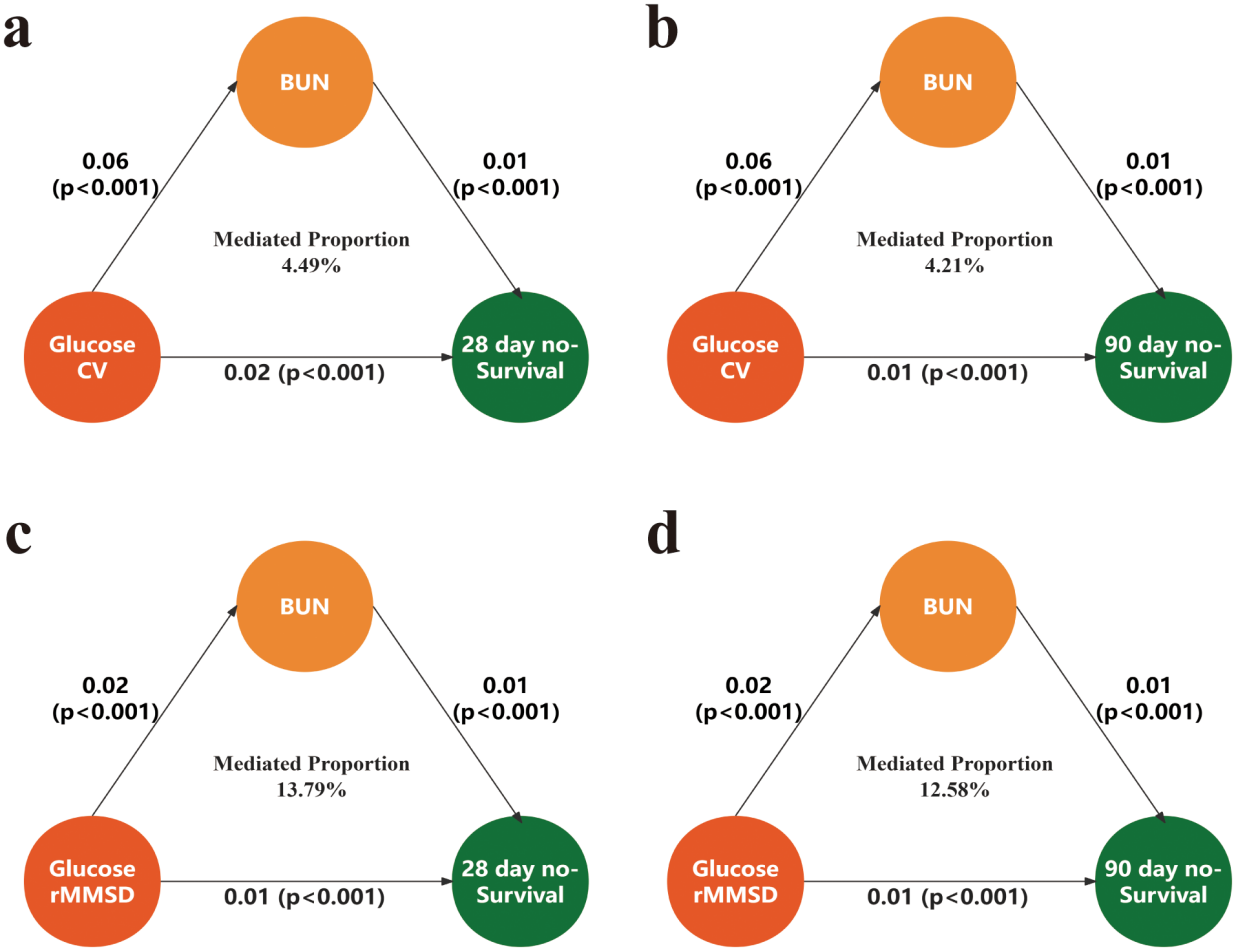
Mediation analysis of the association between glycemic variability and mortality via BUN. **(a)** Mediation of the association between glucose CV and 28-day mortality. **(b)** Mediation of the association between glucose CV and 90-day mortality. **(c)** Mediation of the association between glucose rMSSD and 28-day mortality. **(d)** Mediation of the association between glucose rMSSD and 90-day mortality. CV, coefficient of variation; rMSSD, root mean square of successive differences; BUN, blood urea nitrogen.

After adjusting for confounding variables, the mediation analysis of blood CV and glucose rMSSD with 28-day and 90-day all-cause mortality revealed significant results. For the 28-day non-survival outcome, the mediated proportion for CV was 4.49%, and for rMSSD, it was 13.79%. For 90-day non-survival, the mediated proportion for CV was 4.21%, and for rMSSD, it was 12.58% (**Figure 4**).

### 3.6 ROC curve analysis of predictors for mortality

**Figure 5:**
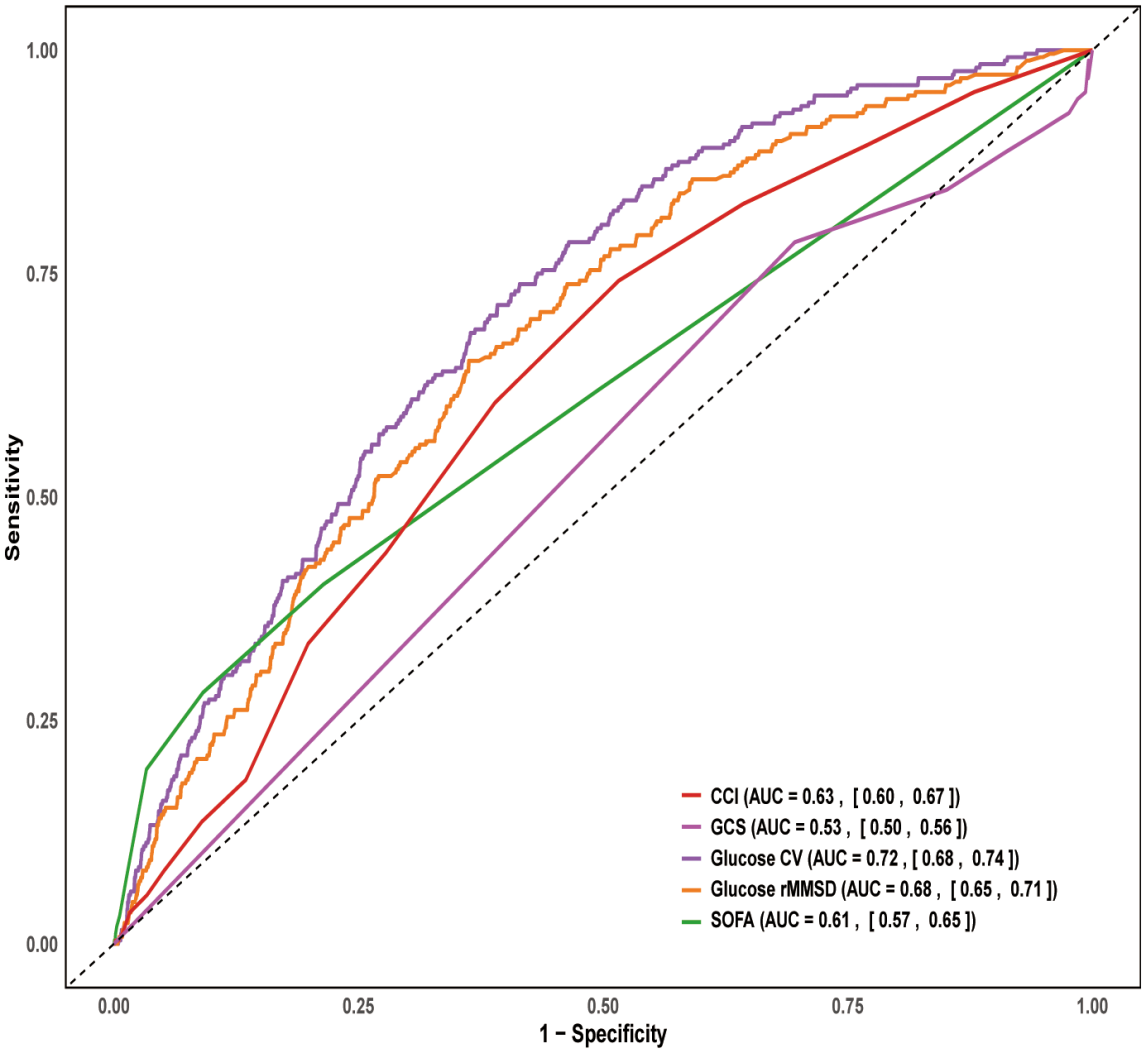
ROC curve for 28-day mortality. ROC, Receiver Operating Characteristic; SOFA, Sequential organ failure assessment; GCS, Glasgow Coma Scale; CCI, Charlson Comorbidity Inde; CV, coefficient of variation; rMSSD, root mean square of successive differences.

The ROC curve analysis for 28-day mortality showed the following AUC values: CCI (AUC = 0.63, 95% CI: 0.60–0.67), GCS (AUC = 0.53, 95% CI: 0.50–0.56), glucose CV (AUC = 0.72, 95% CI: 0.68–0.74), glucose rMSSD (AUC = 0.68, 95% CI: 0.65–0.71), and SOFA (AUC = 0.61, 95% CI: 0.67–0.65). Among these, glucose CV and glucose rMSSD demonstrated the highest predictive ability for 28-day mortality (**Figure 5**).

### 3.7 Feature selection for machine learning models in predicting 28-day all-cause mortality risk

The data were split into training (N = 1,378) and test sets (N = 591) based on predefined criteria. Feature testing was performed between the two groups, and all P-values were greater than 0.05, indicating no significant differences in baseline characteristics between the training and test sets. The number of positive cases for 28-day hospital mortality was 179 (13%) in the training set and 77 (13%) in the test set (**Supplementary Table S3**). Kaplan-Meier curves were generated for both sets, and log-rank tests yielded a P-value of 1 (**Supplementary Figure S2**), suggesting no significant difference between the groups in terms of survival.

**Figure 6:**
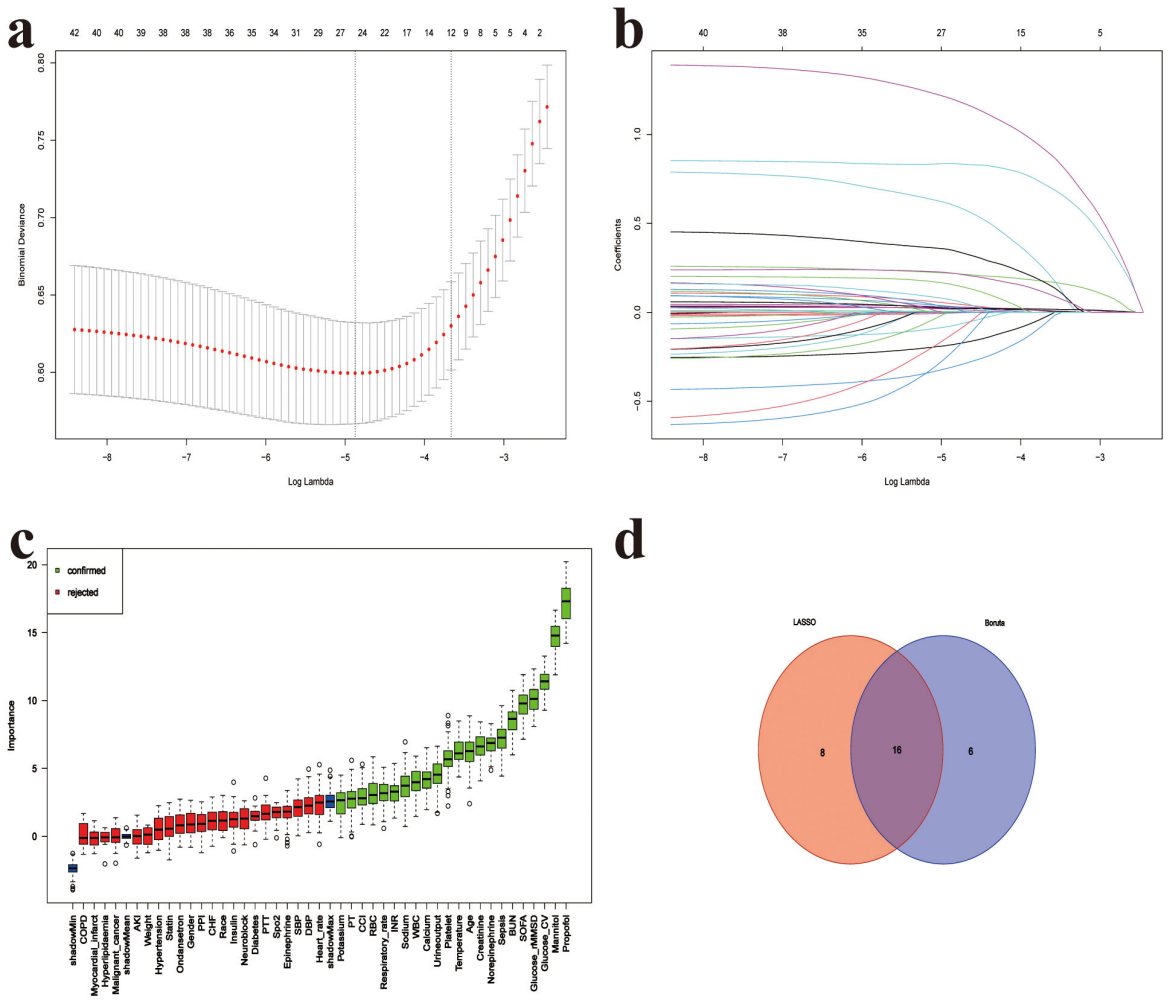
Feature selection for 28-day mortality prediction model. **(a)** Five-fold cross-validation for LASSO logistic regression identifying the optimal value of the regularization parameter (lambda), **(b)** LASSO coefficient profiles of candidate predictors as a function of log(lambda), **(c)** Variable importance ranking based on the Boruta algorithm; green bars represent confirmed important features, red bars indicate rejected ones, **(d)** Venn diagram showing the intersection of features selected by both LASSO and Boruta methods; 16 features were identified by both approaches. SOFA, Sequential organ failure assessment; GCS, Glasgow Coma Scale; CCI, Charlson Comorbidity Index; SpO2, Oxygen saturation; SBP, Systolic blood pressure; DBP, Diastolic blood pressure; AKI, Acute kidney injury; WBC, White blood cell count; RBC, Red blood cell count; Platelet, Platelet count; CV, coefficient of variation; rMSSD, root mean square of successive differences; BUN, blood urea nitrogen; INR, International normalized ratio; PPI, Proton Pump Inhibitor; MV, Mechanical Ventilation; CRRT, Continuous renal replacement therapy.

Based on the predefined feature selection criteria, 24 variables were included in the Lasso model. After applying the Boruta method, 22 variables were selected, with glucose CV and glucose rMSSD being the 3rd and 4th most important variables, respectively, outperforming SOFA and CCI. The final set of 16 variables was derived by taking the intersection of the two methods. These variables include: age, body temperature, SOFA score, CCI, RBC, WBC, sodium, calcium, INR, BUN, norepinephrine, sepsis, propofol, mannitol, glucose CV, and glucose rMSSD (**Figure 6**).

### 3.8 Development of a machine learning prediction model for 28-day all-cause mortality

The training set includes 179 positive cases. After feature selection, 16 variables were identified, which meet the criteria of the Event per Variable principle. We trained the models based on the selected features, including CoxPH, CART, GBM, Ridge, ENet, NN, RSF, and XGBoost, and performed grid search for hyperparameter tuning during the training process.

**Figure 7:**
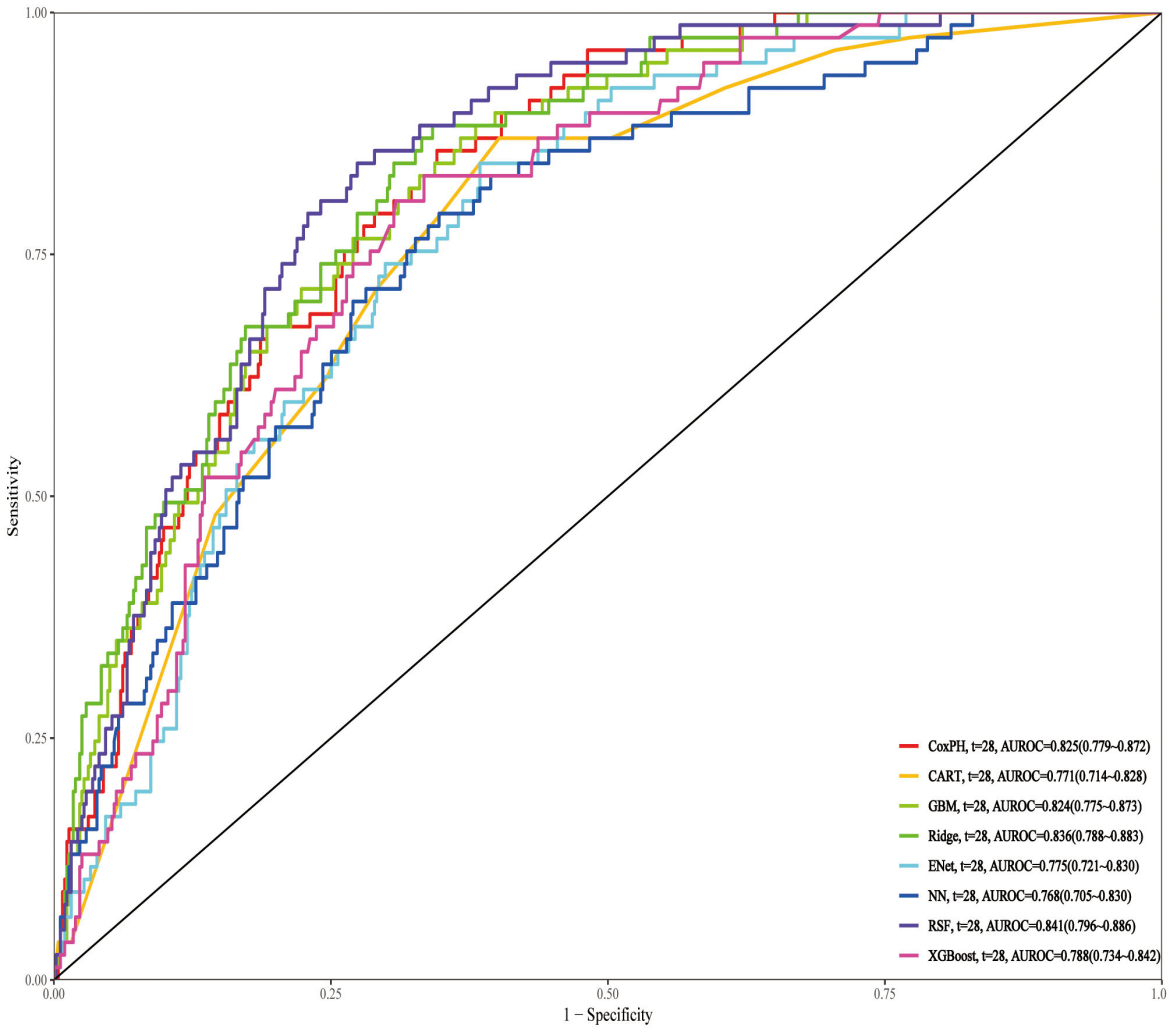
ROC curves of different prediction models for 28-day mortality in the test set. ROC, Receiver Operating Characteristic; RSF, Random Survival Forest; Ridge, Ridge regression; CoxPH, Cox proportional hazards model; GBM, Gradient Boosting Machine; XGBoost, Extreme Gradient Boosting; CART, Classification and Regression Trees; Enet, Elastic Net; NN, Neural Network.

On the test set, RSF achieved the highest AUROC of 0.841, outperforming all other models, including Ridge (0.836) and CoxPH (0.825). Other models, such as GBM (0.824), XGBoost (0.788), CART (0.771), ENet (0.775), and NN (0.768), performed relatively lower (**Figure 7**). Further evaluation with decision curve analysis and calibration curves confirmed that RSF was the optimal model, showing the highest net benefit and well-calibrated predicted probabilities, making it the best model for predicting 28-day all-cause mortality (**Supplementary Figure S3**).

### 3.9 SHAP interpretation of the RSF model

**Figure 8:**
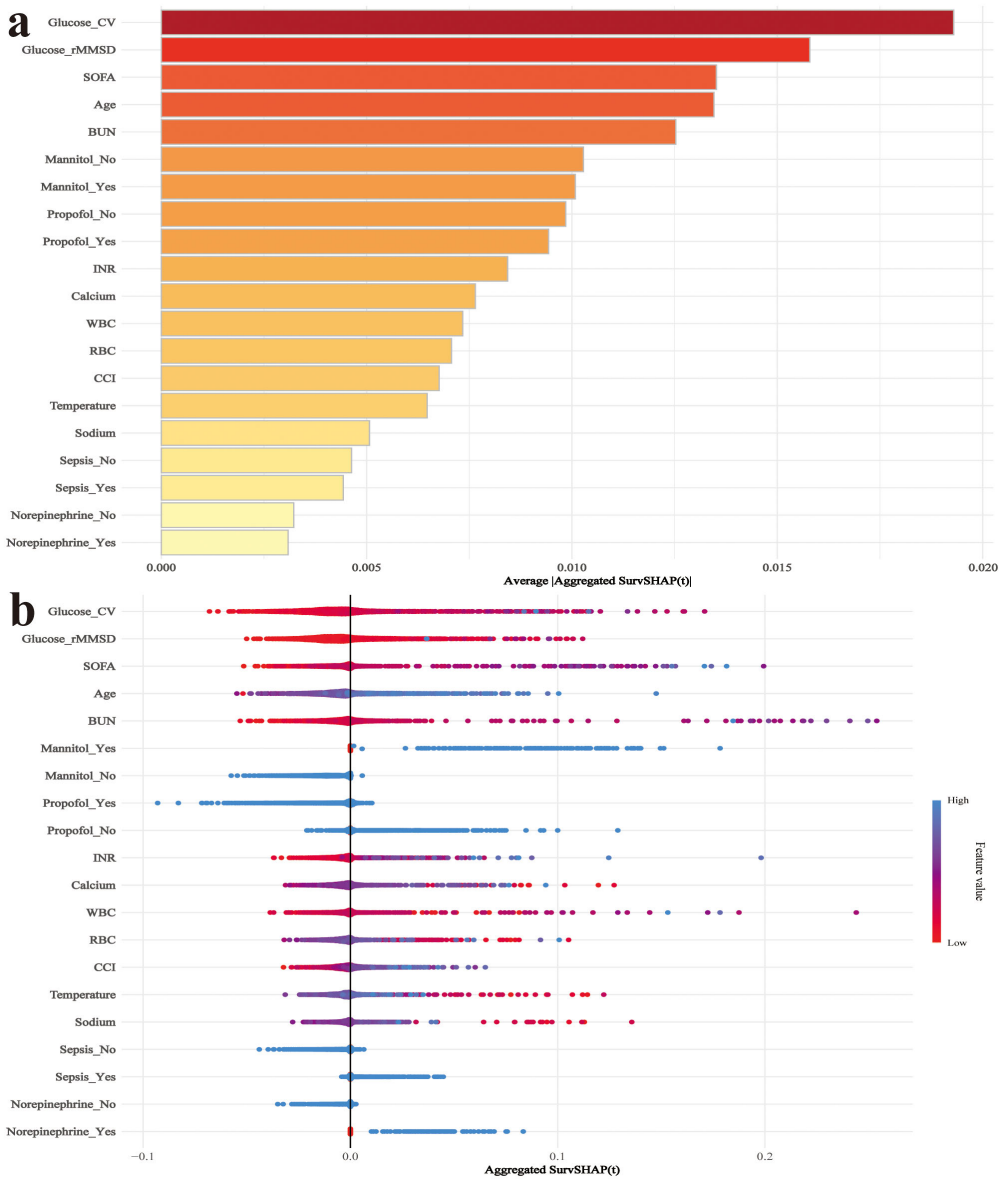
SHAP analysis for interpretation of the RSF model predicting 28-day mortality. **(a)** SHAP summary plot showing the importance ranking of selected features based on their mean absolute SHAP values. **(b)** SHAP dependence plot displaying the distribution of SHAP values for each feature. Colors represent feature values, with red indicating high values and blue indicating low values. WBC, White blood cell count; RBC, Red blood cell count; Platelet, Platelet count; CV, coefficient of variation; rMSSD, root mean square of successive differences; INR, International normalized ratio.

The SHAP summary plot of the RSF model reveals that glucose CV and glucose rMSSD are the two most important variables influencing mortality risk in post-brain surgery patients (**Figure 8**). Both features show a strong positive association with SHAP values, indicating that higher glucose variability increases the predicted risk of death. To enhance clinical usability, we deployed the RSF model on a publicly accessible web platform (https://docterge.shinyapps.io/Craniotomy/.). This tool allows users to input individual patient data and receive a personalized prediction of 28-day mortality risk. In addition to the probability output, the tool provides individualized SHAP visualizations to help interpret which clinical features contributed most to the prediction. An example of the prediction interface and interpretability output is provided in **Supplementary Figure S4**.

## 4 Discussion

This study examined the association between blood glucose fluctuations and mortality risk in post-craniotomy patients. glucose CV and glucose rMSSD were significantly associated with both 28-day and 90-day mortality, with the highest quantiles of both variables showing the strongest associations. Glucose CV achieved an AUC of 0.72, significantly outperforming SOFA, GCS, and CCI. In addition, the mortality prediction model incorporating glucose variability demonstrated strong discriminative performance, with the RSF model reaching an AUC of 0.841. SHAP interpretation indicated that glucose CV and glucose rMSSD were the most important predictors of 28-day mortality. The RSF model has been successfully deployed on a web platform, providing a convenient tool for real-time risk prediction and valuable insights for glucose management in clinical practice.

Previous studies have consistently demonstrated that glycemic abnormalities are associated with poor outcomes in critically ill patients. In a large cohort of septic patients, stress hyperglycemia was independently linked to increased mortality, highlighting the detrimental impact of acute glucose dysregulation in inflammatory states (18). Similarly, in patients with coronary artery disease, higher GV was significantly associated with adverse cardiovascular events and all-cause mortality (17). GV has also been implicated in the development of ventricular arrhythmias in ICU patients, further supporting its role as a marker of physiological instability (19). In surgical populations, several studies have identified GV as a predictor of postoperative complications and mortality. For example, GV was associated with increased mortality in patients undergoing aortic valve replacement and linked to postoperative atrial fibrillation in cardiac surgery patients (20, 21). Despite this growing body of evidence, neurosurgical patients remain underrepresented in the literature. Given their heightened vulnerability to secondary brain injury and distinct neuroendocrine responses to surgical stress, this population warrants targeted investigation (22). Our study addresses this gap by evaluating both CV and rMSSD in relation to postoperative mortality in cranial surgery patients, offering a novel perspective on the prognostic implications of short- and mid-term glycemic variability in neurocritical care.

Patients undergoing cranial surgery represent a high-risk subgroup in critical care, characterized by disrupted cerebral autoregulation, compromised blood–brain barrier (BBB), and reduced tolerance to secondary systemic insults (23). In this context, blood glucose fluctuations—rapid oscillations beyond physiological ranges—may function not merely as markers of stress, but as active pathophysiological drivers of poor outcomes (24). At the cerebral level, the postoperative brain is highly sensitive to osmotic shifts due to surgical disruption of the BBB (25). Abrupt glucose changes alter serum osmolality, promoting fluid shifts that exacerbate cerebral edema (26). In neurosurgical patients, where intracranial compliance is often reduced, even modest volume increases may elevate intracranial pressure, reduce cerebral perfusion pressure, and restrict oxygen and substrate delivery to metabolically vulnerable brain regions (27, 28). Moreover, the brain’s near-exclusive dependence on glucose renders it highly vulnerable to both hyperglycemia and hypoglycemia (29). Hyperglycemia promotes mitochondrial dysfunction, reactive oxygen species generation, anaerobic glycolysis, and lactic acidosis—worsening neuronal injury in peri-infarct zones (30, 31). Hypoglycemia, conversely, deprives neurons of ATP, resulting in ion pump failure, glutamate excitotoxicity, and calcium-mediated apoptosis (32). When such extremes occur in close proximity, they may trigger recurrent ischemia-reperfusion cycles that amplify neuronal death (33). Glucose fluctuations also provoke endothelial activation and systemic inflammation (34). Oscillating glucose levels stimulate NF-κB signaling, elevate cytokines (e.g., IL-6, TNF-α), and impair nitric oxide-mediated vasodilation (34, 35). This proinflammatory, vasoconstrictive milieu promotes microthrombosis, capillary leakage, and impaired cerebral microcirculation (35). Beyond the CNS, blood glucose fluctuations are associated with multi-organ stress responses. In patients undergoing major surgery, blood glucose fluctuations are independently associated with acute kidney injury, cardiac dysfunction, and sepsis, each of which may compromise systemic perfusion and exacerbate cerebral hypoxia (36). Together, these mechanisms support blood glucose fluctuations as not passive markers, but active contributors to mortality in post-craniotomy patients.

An intriguing finding in this study was the identification of BUN as a partial mediator in the relationship between blood glucose fluctuations and mortality following craniotomy. Mediation analysis revealed that BUN accounted for 4.49% of the association between glucose CV and 28-day mortality, and 13.79% for glucose rMSSD. Similar trends were observed for 90-day outcomes, with mediated proportions of 4.21% and 12.58%, respectively. Although the proportion mediated was modest, the consistency and statistical significance across time points suggest a biologically plausible pathway linking glycemic instability to adverse outcomes through renal dysfunction. BUN, a marker of nitrogen metabolism and renal perfusion, may reflect subclinical renal injury triggered by glucose fluctuations. Repeated episodes of hyperglycemia can induce glomerular hyperfiltration, oxidative stress, and endothelial damage (37). Furthermore, glucose variability has been associated with systemic inflammation and sympathetic activation, both of which can impair renal autoregulation and contribute to elevated BUN (34). In critically ill neurosurgical patients, elevated BUN may serve as both a marker and mediator of physiological stress. However, given the relatively low mediated proportions, particularly for CV (4.21–4.49%), these findings should be interpreted with caution, and BUN should be regarded as a secondary rather than a primary mechanistic pathway.

The findings of this study have important implications for the postoperative management of patients undergoing cranial surgery. Glycemic fluctuations, traditionally viewed as an epiphenomenon of critical illness, should be reconsidered as modifiable contributors to poor neurological outcomes. Their independent association with both short-term and long-term mortality, along with mechanistic links involving cerebral, renal, and systemic pathways, argue for a more proactive and structured therapeutic approach. First, these data highlight the limitations of relying solely on single-point glucose values or mean glycemic indices, which may obscure clinically significant variability. Continuous glucose monitoring (CGM) systems, now increasingly available in critical care settings, may offer real-time visibility into dynamic glucose trends, facilitating earlier detection of deleterious fluctuations and more precise glycemic control. Second, our results suggest that maintaining glycemic stability—rather than pursuing tight control—may be more beneficial in neurosurgical patients. Protocols that minimize glucose variability, such as structured insulin titration algorithms and threshold-based interventions, could reduce the risk of secondary brain injury while avoiding hypoglycemia. Third, the identification of BUN as a partial mediator suggests the utility of integrated organ monitoring. Early rises in BUN, particularly in patients with marked glycemic variability, may serve as early warning indicators of physiological decompensation. Finally, these findings support the development of multidisciplinary care pathways that engage endocrinology, nephrology, and neurocritical care teams. Such pathways should include practical components like early CGM initiation post-craniotomy, predefined thresholds for intervention, and the integration of GV metrics (e.g., CV, rMSSD) into ICU monitoring dashboards or alert systems. Future prospective studies are needed to validate these approaches and assess whether interventions targeting GV can lead to improved clinical outcomes in this high-risk population.

This study has several limitations. First, it was based on data from a single academic medical center, which may limit the generalizability of the findings to other settings. Second, as a retrospective observational study, causal inference cannot be established, and residual confounding remains possible—particularly from unmeasured factors such as preoperative glucose control and nutritional status. Furthermore, the absence of a standardized glucose monitoring protocol in the ICU may have introduced heterogeneity in the timing and frequency of glucose measurements, potentially affecting the accuracy of glycemic variability estimates. For instance, patients receiving more frequent glucose checks—often due to clinical instability or active insulin titration—may have artificially inflated GV metrics due to closer monitoring. Conversely, less frequent testing in more stable patients could lead to underestimation of variability. Additionally, although insulin use was adjusted for in the multivariable analysis, detailed information on insulin administration protocols, including dosage, delivery mode (e.g., bolus vs. infusion), and responsiveness to glycemic excursions, was unavailable. These therapeutic factors may themselves influence both the degree of GV and the associated clinical outcomes, representing an important source of residual confounding. Future prospective studies with standardized glucose monitoring schedules and detailed insulin management data are warranted to address these limitations.

## 5 Conclusion

This study is the first to systematically assess the prognostic relevance of blood glucose fluctuations in patients following craniotomy. Both glucose CV and glucose rMSSD were independently associated with 28-day and 90-day mortality, exhibited non-linear relationships, and were partially mediated through BUN. GV outperformed traditional clinical scores such as SOFA and CCI in mortality risk prediction. A machine learning model incorporating glucose variability demonstrated high discriminative performance and has been successfully deployed on a clinical web-based platform. These findings support GV as a modifiable risk factor and underscore its potential role in postoperative risk stratification and management for neurosurgical patients. Further prospective, multicenter studies are warranted to validate these findings and explore whether targeted interventions on GV can improve clinical outcomes.

## Data Availability

The original contributions presented in the study are included in the article/**Supplementary Material**. Further inquiries can be directed to the corresponding author.
