# Supplementary material for: Association between the postoperative glycemic variability and mortality after craniotomy: a retrospective cohort study and development of a mortality prediction model
Source: Front Endocrinol (Lausanne). 2025 Jul 17;16:1613662. doi: 10.3389/fendo.2025.1613662 (PMC12310502; doi:10.3389/fendo.2025.1613662)
Supplement: Supplementary file 1 [file DataSheet1.docx]

**Supplementary Material**

Table S1 Missing values in variables.

Table S2 Univariable cox regression analysis for 28-day in-hospital all-cause mortality.

Table S3 Characteristics of patient in training set and validation set.

Fig. S1 Study cohort inclusion flowchart

Fig. S2 Kaplan–Meier survival curves for mortality in the training and test sets.

Fig. S3 (a) Calibration curves for multiple models in the validation Set. (b) Decision curves for multiple models in the validation set.

Fig. S4 Web-based RSF prediction tool for 28-day mortality after craniotomy.

**TableS1 missing values in variables**

| **Column** | **Missing Percentage (%)** |
| --- | --- |
| Weight | 9.5479939 |
| PTT | 9.4464195 |
| INR | 8.8877603 |
| PT | 8.8369731 |
| Calcium | 2.5393601 |
| Urine output | 1.26968 |
| RBC | 0.8633824 |
| WBC | 0.8125952 |
| Platelet | 0.8125952 |
| BUN | 0.8125952 |
| Sodium | 0.761808 |
| Potassium | 0.761808 |
| Creatinine | 0.761808 |
| Respiratory rate | 0.5586592 |
| Heart rate | 0.1015744 |
| SBP | 0.1015744 |
| DBP | 0.1015744 |
| Temperature | 0.1015744 |
| Spo2 | 0.1015744 |
| SOFA | 0.1015744 |
| GCS | 0.1015744 |

Abbreviation: SOFA: Sequential organ failure assessment, GCS: Glasgow Coma Scale, SBP: Systolic blood pressure, DBP: Diastolic blood pressure, WBC: White blood cell count, RBC: Red blood cell count, Platelet: Platelet count, INR: International normalized ratio

**Table S2. Univariable cox regression for 28-day all-cause mortality**

| **Variables** | **HR (95%CI)** | ***P*** |
| --- | --- | --- |
| Gender |  |  |
| Female | 1.00 (Reference) |  |
| Male | 1.02 (0.80–1.30) | 0.889 |
| Race |  |  |
| Other | 1.00 (Reference) |  |
| White | 0.47 (0.37– 0.60) | **<0.001** |
| Age | 1.02 (1.01–1.03) | **<0.001** |
| Weight | 1.00 (0.99–1.00) | 0.596 |
| Respiratory rate | 1.07 (1.05–1.09) | **<0.001** |
| Temperature | 0.76 (0.66–0.87) | **<0.001** |
| Spo2 | 1.13 (1.06–1.21) | **<0.001** |
| SOFA | 1.35 (1.27–1.44) | **<0.001** |
| GCS | 0.95 (0.89–1.00) | 0.063 |
| CCI | 1.12 (1.08–1.16) | **<0.001** |
| Congestive heart failure |  |  |
| No | 1.00 (Reference) |  |
| Yes | 2.23 (1.56–3.18) | **<0.001** |
| Malignant cancer |  |  |
| No | 1.00 (Reference) |  |
| Yes | 0.48 (0.32–0.73) | **<0.001** |
| Diabetes |  |  |
| No | 1.00 (Reference) |  |
| Yes | 1.81 (1.38–2.37) | **<0.001** |
| Hypertension |  |  |
| No | 1.00 (Reference) |  |
| Yes | 2.07 (1.57–2.72) | **<0.001** |
| RBC | 0.64 (0.53–0.77) | **<0.001** |
| Platelet | 0.99 (0.99–0.99) | **0.004** |
| INR | 2.52 (1.83–3.47) | **<0.001** |
| PT | 1.01 (1.01–1.02) | **0.024** |
| PTT | 1.01 (1.01 –1.02) | **<0.001** |
| BUN | 1.02 (1.02–1.03) | **<0.001** |
| Creatinine | 1.28 (1.17–1.39) | **<0.001** |
| Urine output | 1.00 (1.00–1.00) | 0.765 |
| Epinephrine |  |  |
| No | 1.00 (Reference) |  |
| Yes | 3.45 (1.28–9.26) | **0.014** |
| Norepinephrine |  |  |
| No | 1.00 (Reference) |  |
| Yes | 3.50 (2.59–4.72) | **<0.001** |
| Insulin |  |  |
| No | 1.00 (Reference) |  |
| Yes | 2.08 (1.60–2.69) | **<0.001** |
| Statin |  |  |
| No | 1.00 (Reference) |  |
| Yes | 1.28 (0.97–1.70) | 0.078 |
| Ondansetron |  |  |
| No | 1.00 (Reference) |  |
| Yes | 0.58 (0.45–0.75) | **<0.001** |
| AKI |  |  |
| No | 1.00 (Reference) |  |
| Yes | 3.09 (2.19–4.36) | **<0.001** |
| Sepsis |  |  |
| No | 1.00 (Reference) |  |
| Yes | 3.24 (2.47–4.25) | **<0.001** |
| Neuroblock |  |  |
| No | 1.00 (Reference) |  |
| Yes | 2.64 (1.40–4.96) | **0.003** |
| Propofol |  |  |
| No | 1.00 (Reference) |  |
| Yes | 4.96 (3.69–6.66) | **<0.001** |
| Mannitol |  |  |
| No | 1.00 (Reference) |  |
| Yes | 3.47 (2.65–4.54) | **<0.001** |

Abbreviation: SOFA: Sequential organ failure assessment, GCS: Glasgow Coma Scale, CCI: Charlson Comorbidity Index, SpO2: Oxygen saturation, RBC: Red blood cell count, INR: International normalized ratio, PT: prothrombin time (PT), PTT: activated partial thromboplastin time, BUN: blood urea nitrogen, AKI: acute kidney injury.

**Table S3 baseline characteristics of the training and test sets**

| **Characteristic** | **Overall**  N = 1,969 | **Train**  N = 1,378 | **Test**  N = 591 | **p-value** |
| --- | --- | --- | --- | --- |
| Age (year) | 63.60 (50.18, 74.16) | 64.01 (50.97, 74.07) | 62.40 (48.21, 74.31) | 0.234 |
| Temperature (℃) | 36.72 (36.44, 37.06) | 36.67 (36.39, 37.00) | 36.72 (36.44, 37.10) | **0.027** |
| SOFA | 1.00 (0.00, 1.00) | 1.00 (0.00, 1.00) | 1.00 (0.00, 1.00) | 0.933 |
| CCI | 4.00 (2.00, 6.00) | 4.00 (2.00, 6.00) | 4.00 (2.00, 6.00) | 0.462 |
| RBC (10^9^/L) | 3.97 (3.53, 4.37) | 3.97 (3.53, 4.37) | 3.95 (3.53, 4.35) | 0.740 |
| WBC (10^9^/L) | 12.10 (9.00, 15.40) | 12.10 (8.90, 15.50) | 12.10 (9.20, 15.30) | 0.648 |
| Sodium (mmol/L) | 140.00 (137.00, 142.00) | 140.00 (137.00, 142.00) | 140.00 (137.00, 142.00) | 0.640 |
| Calcium (mg/dL) | 8.50 (8.10, 8.90) | 8.50 (8.10, 8.90) | 8.50 (8.10, 8.90) | 0.906 |
| INR | 1.10 (1.10, 1.20) | 1.10 (1.10, 1.20) | 1.10 (1.10, 1.20) | 0.503 |
| BUN (mg/dL) | 14.00 (10.00, 19.00) | 14.00 (10.00, 19.00) | 14.00 (11.00, 19.00) | 0.412 |
| Norepinephrine, n (%) | 159 (8%) | 105 (8%) | 54 (9%) | 0.257 |
| Sepsis, n (%) | 891 (45%) | 615 (45%) | 276 (47%) | 0.398 |
| Propofol, n (%) | 862 (44%) | 596 (43%) | 266 (45%) | 0.471 |
| Mannitol, n (%) | 239 (12%) | 164 (12%) | 75 (13%) | 0.623 |
| Glucose CV (%) | 14.97 (9.69, 21.70) | 15.01 (9.65, 22.11) | 14.97 (9.72, 21.05) | 0.647 |
| Glucose rMMSD | 23.00 (14.80, 37.00) | 23.30 (14.65, 37.45) | 22.51 (15.23, 34.77) | 0.693 |
| Time (day) | 28.00 (28.00, 28.00) | 28.00 (28.00, 28.00) | 28.00 (28.00, 28.00) | 0.985 |
| 28-day hospital Mortality  , n (%) | 256 (13%) | 179 (13%) | 77 (13%) | 0.981 |
| Abbreviation: WBC: White blood cell count, RBC: Red blood cell count, Platelet: Platelet count, CV: coefficient of variation, rMMSD: root mean square of successive differences, INR: International normalized ratio | | | | |

**
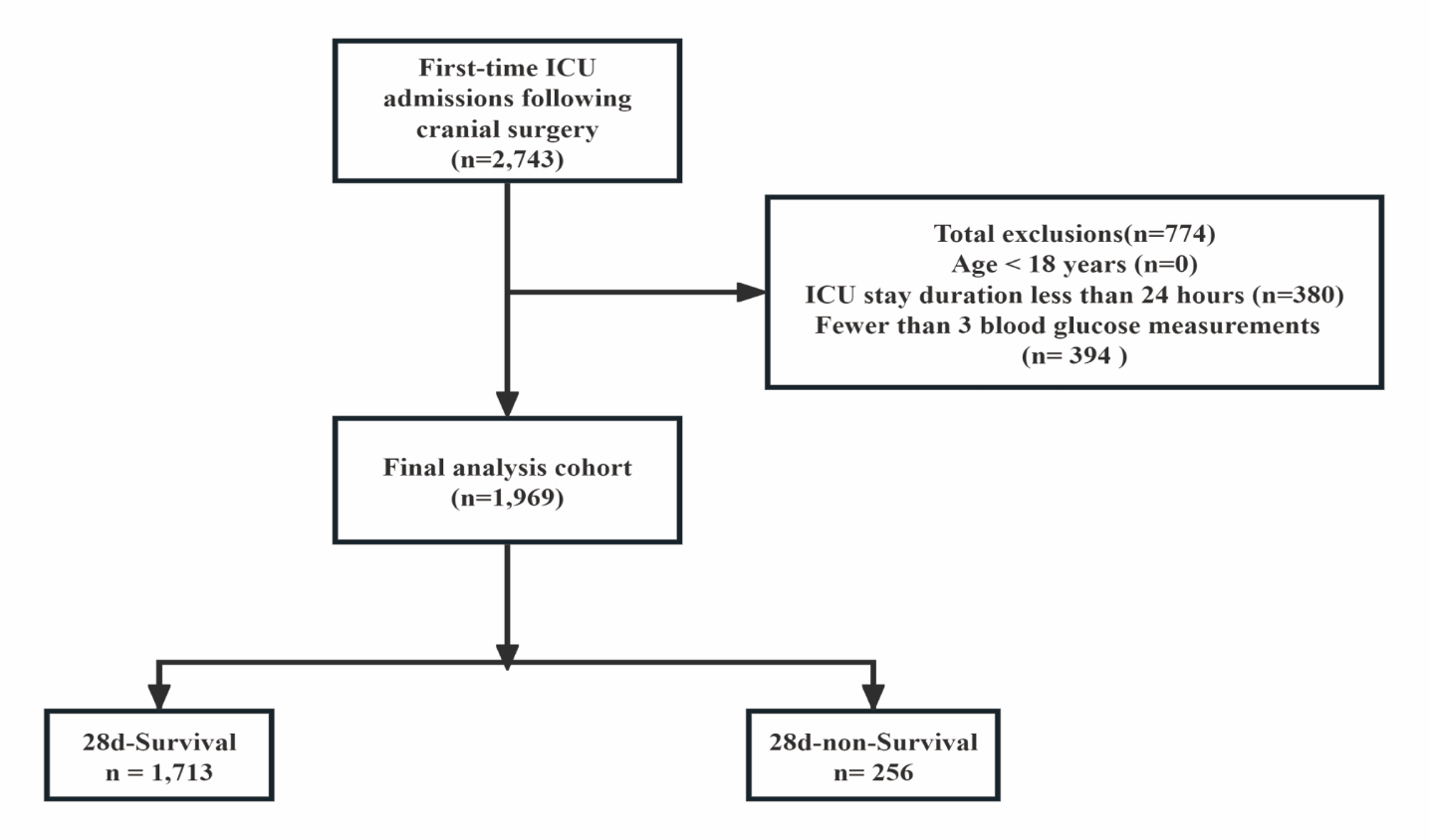
**

**Fig. S1 (A) Decision curves for multiple models in the validation set. (B) Calibration curves for multiple models in the validation Set.**

**
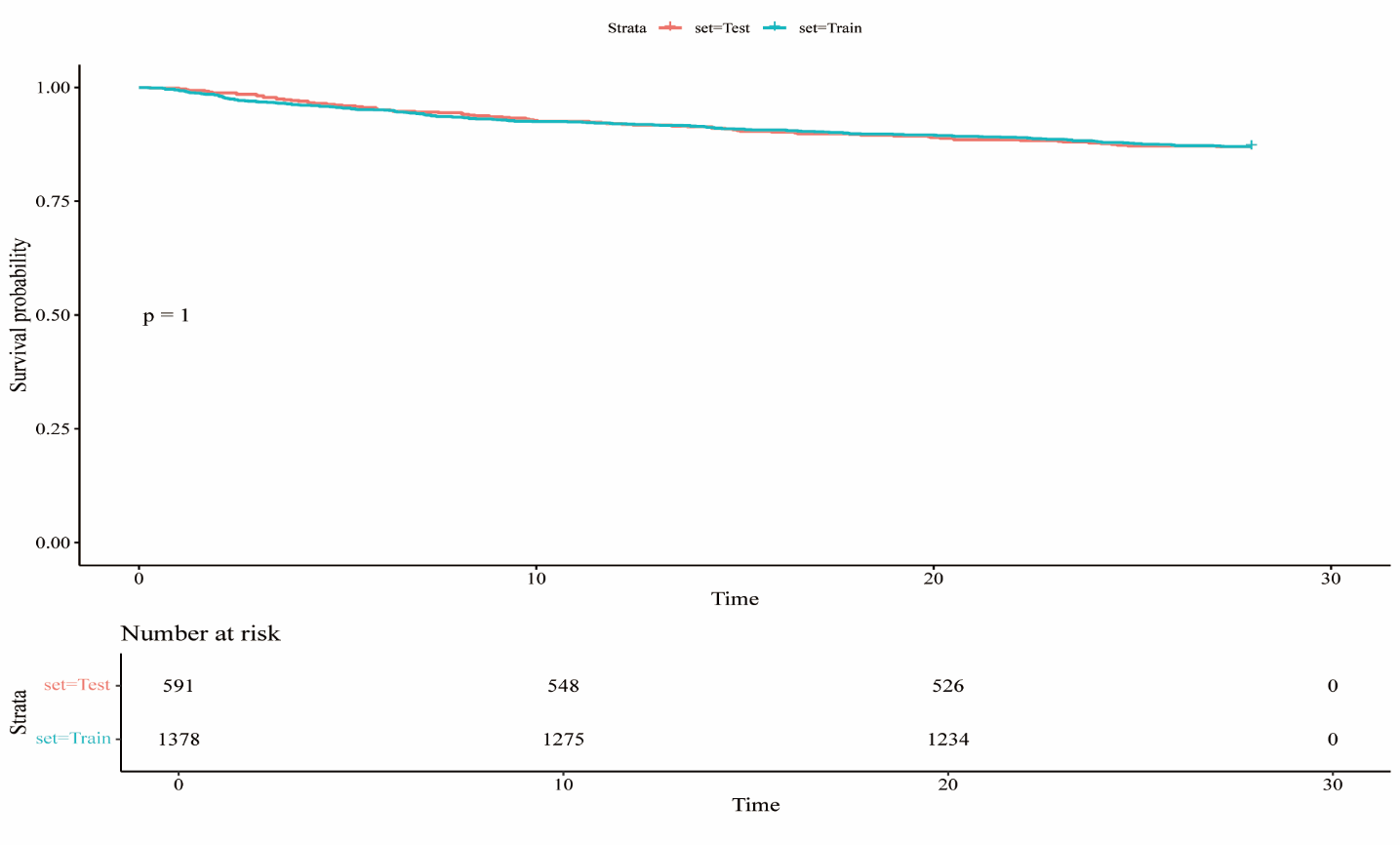
**

**Fig. S2 Kaplan–Meier survival curves for mortality in the training and test sets.**

**
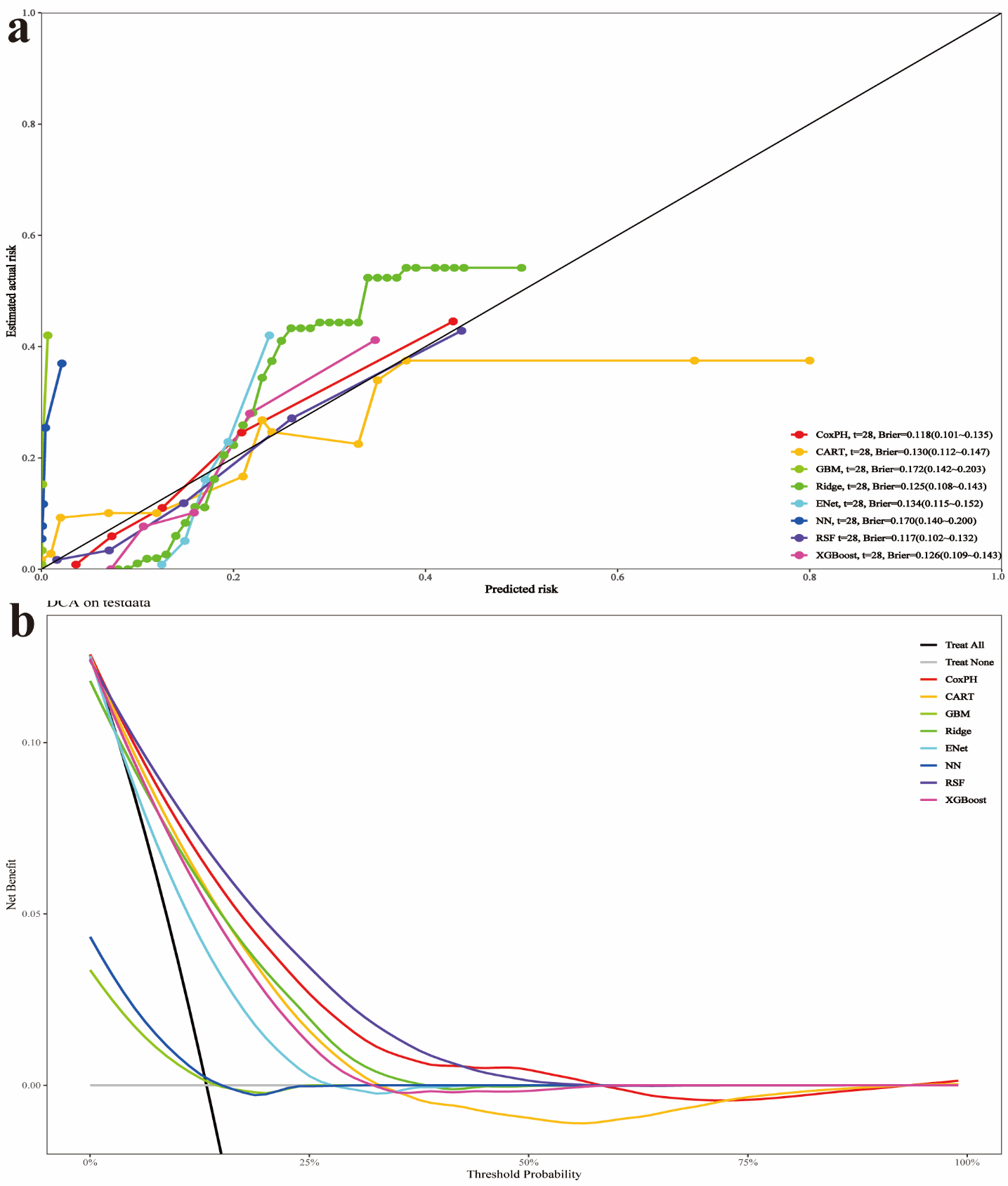
**

**Fig. S3 (a) Calibration curves for multiple models in the validation Set. (b) Decision curves for multiple models in the validation set.**

**
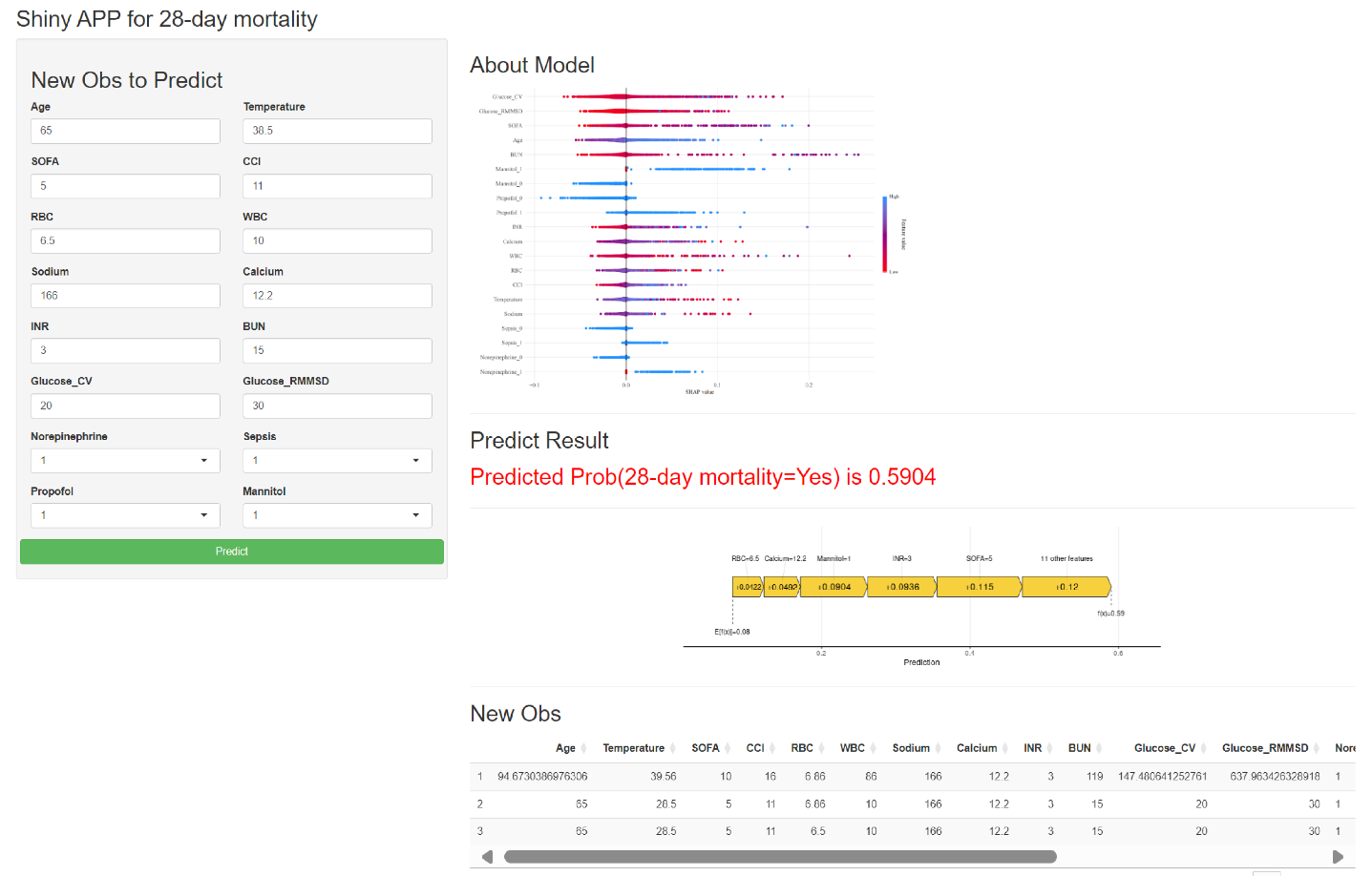
**

**Fig. S4 Web-based RSF prediction tool for 28-day mortality after craniotomy.** The online tool allows clinicians to input individual patient data—including age, SOFA score, CCI, selected laboratory values (e.g., RBC, WBC, INR, BUN), and glucose variability metrics (CV and rMSSD)—into a simple interface. Upon submission, the system returns a predicted probability of 28-day mortality, displayed numerically (e.g., 0.5904) and visualized using SHAP values. The SHAP bar plot indicates how each variable contributes positively or negatively to the risk prediction for that individual, helping clinicians interpret the model's reasoning. This tool is designed for research and educational purposes, and may support real-time risk stratification in neurosurgical ICU settings. Web platform URL: https://docterge.shinyapps.io/Craniotomy/
